# Supplementary material for: Interpreting nanovoids in atom probe tomography data for accurate local compositional measurements
Source: Nat Commun. 2020 Feb 24;11:1022. doi: 10.1038/s41467-020-14832-w (PMC7039975; doi:10.1038/s41467-020-14832-w)
Supplement: Supplementary file 1 — Supplementary Information [file 41467_2020_14832_MOESM1_ESM.pdf]

Supplementary Information

Interpreting Nano-voids in Atom Probe Tomography Data for Accurate Local Compositional Measurements

Wang et al.

Supplementary Note 1: Characterization of chemical segregation near nano-voids in NiCoCr using energy dispersive X-ray spectroscopy (EDS)

EDS analysis was conducted for the NiCoCr samples using scanning transmission electron microscope (STEM). Supplementary Figure 1 shows the obtained high angle annular dark field (HAADF) image, corresponding EDS element mapping, and a linear concentration profile. Note that although voids exhibit as dark spheres in the HAADF image (Supplementary Figure. 1a), the element segregation regions around the voids are not spherical (Supplementary Figure 1b). Two factors may contribute to this phenomenon. First, since some voids are spatially close to each other, the element concentration gradients introduced by each void may interact with each other. Second, the 3D samples are projected onto a 2D plane during the EDS mapping, the segregation regions from different voids through the TEM lamella thickness can be overlapped because of the projection. Both factors contribute to the non-spherical segregation regions shown in Supplementary Figure. 1b.

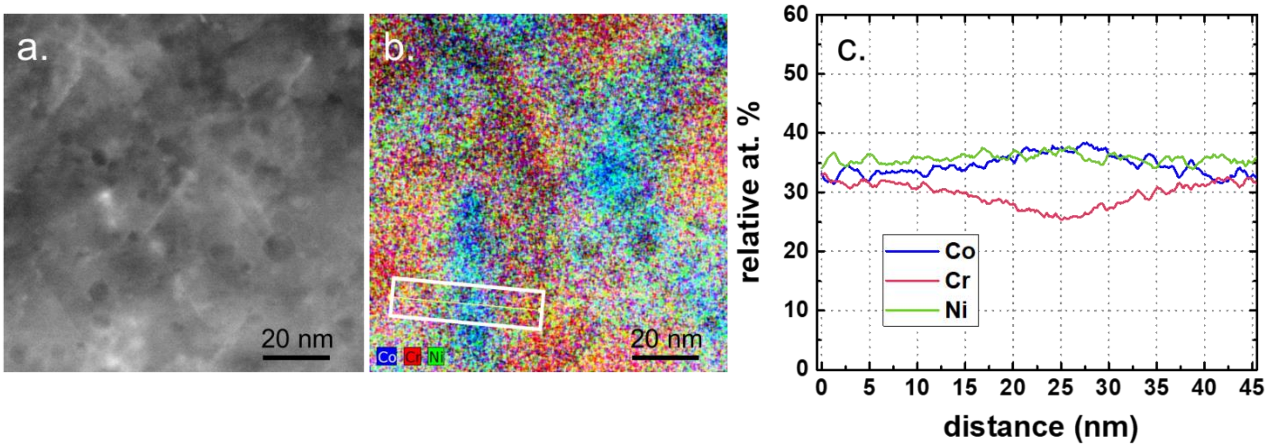

Supplementary Figure 1 Segregation measurements near voids using STEM-based EDS. (a) STEM-HAADF image showing bubbles as dark spherical regions. (b) Corresponding EDS mapping. (c) Concentration profile obtained from a line scan showing segregation near nano-sized voids in NiCoCr. The scanned region is marked by a white rectangle in (b).

Supplementary Note 2: Analysis of atomic density changes near helium bubbles in Ni and NiFe using atom probe tomography (APT)

Pure Ni and NiFe alloys were irradiated by the same 200 keV He ion in the same condition as the NiCoCr sample. Similar APT analyses were performed. Supplementary Figures. 2a and 2b are the obtained one-dimensional (1D) density profile and two-dimensional (2D) density contours near a void in Ni, and Supplementary Figures. 2c and 2d are similar plots near a void in NiFe. In these figures, the reduced density

is defined as the local atomic density divided by the average atomic density in the matrix. The 1D profiles are calculated using a cylindrical ROI with a 5 nm diameter passing through the void in the z-direction of the APT dataset. The 2D contours are calculated using 30 nm×30 nm×3 nm rectangular ROIs slicing the void. In the 1D density profiles, the same λ shape as shown in the NiCoCr sample can be observed near the void. Cone-shaped high-density regions in 2D contour plots introduced by nanosized voids are found in Ni and NiFe as well, which are similar to the local density variations observed near voids in NiCoCr.

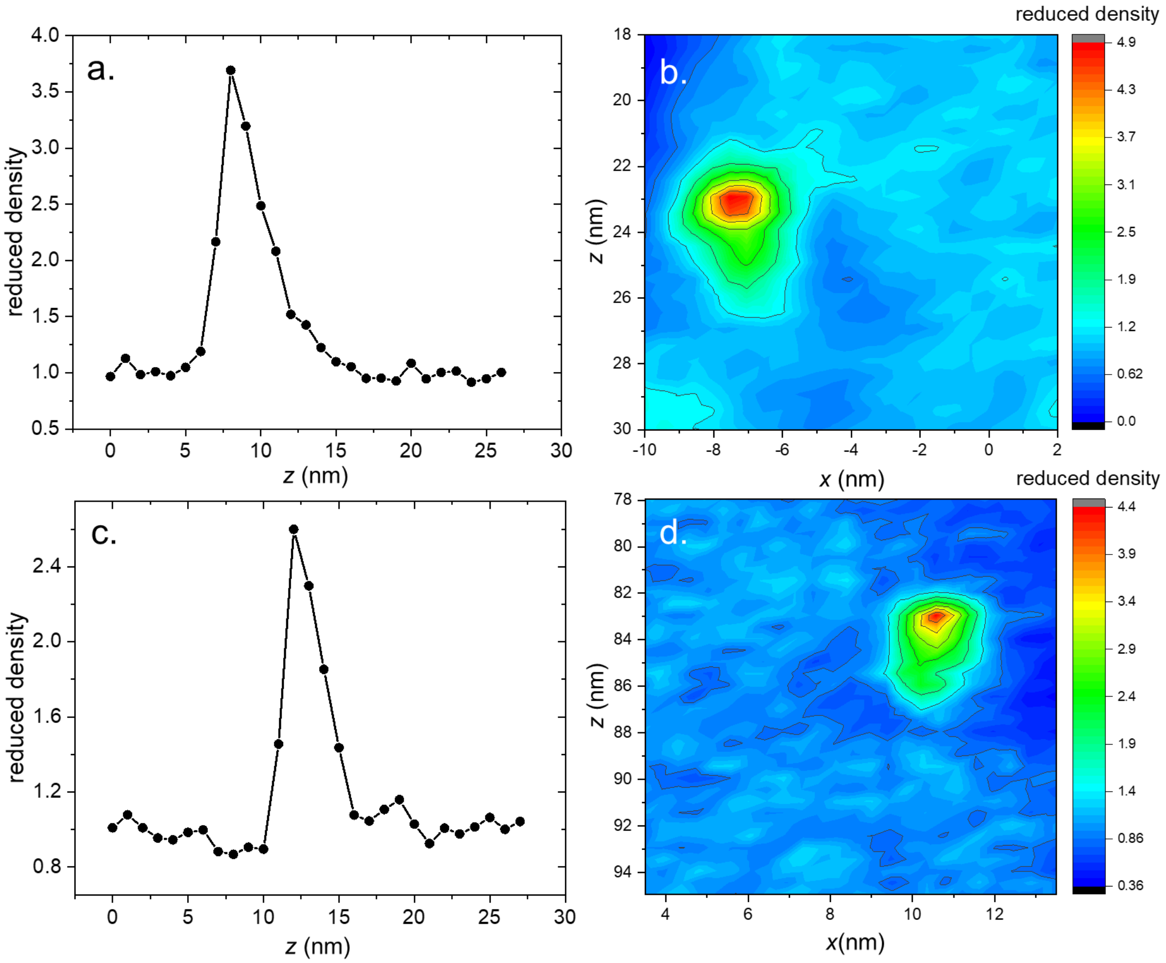

Supplementary Figure 2 Local density variations near voids in Ni and NiFe samples. (a and b) 1D profile and 2D contour plot of local density variations in Ni. (c and d) 1D profile and 2D contour plot of local density variations in NiFe.

Supplementary Note 3: Analysis of changes in local atomic density near a void based on APT analysis using voltage mode

To examine the possible effects of APT running conditions on the local atomic density near nanosized voids, an APT specimen was prepared using the same NiCoCr bulk sample and analyzed using the same CAMECA LEAP 4000X HR system in voltage mode. Fig. S3 shows the obtained 1D profile and 2D contour plots of local density variations near a void, which are very similar to those obtained in the APT specimen run in laser mode. Therefore, it is demonstrated that the changes in local density and chemistry are introduced by the special geometry of the void and are independent of the APT running conditions.

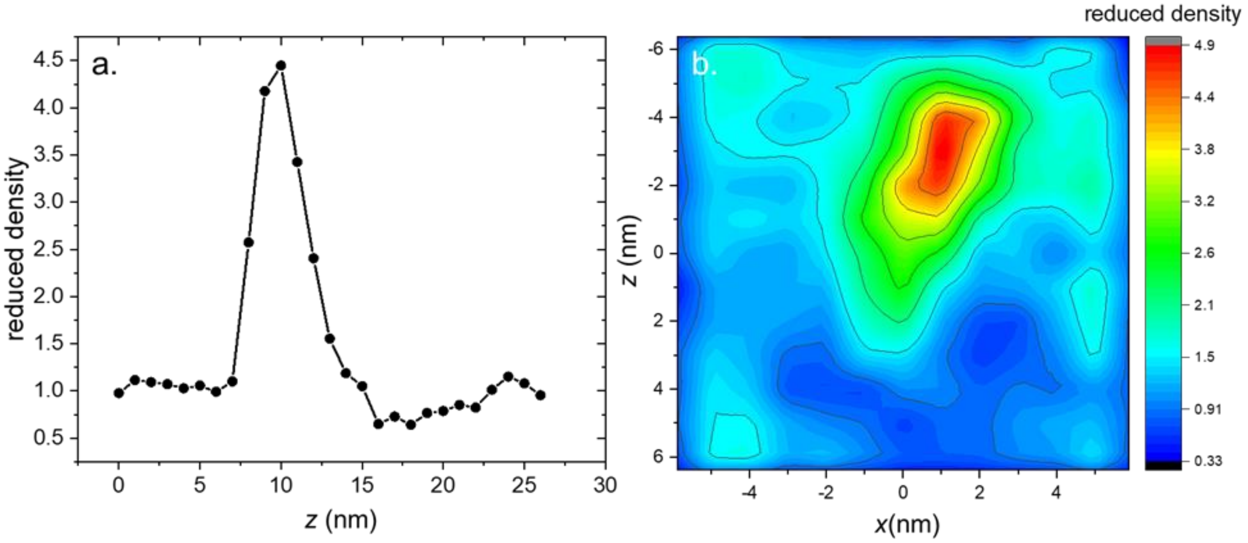

Supplementary Figure 3 Local density variations near a void in the APT reconstruction obtained using voltage mode from the NiCoCr sample. (a) 1D profile and (b) 2D contour plot of local density variations.

Supplementary Note 4: APT needle morphology and electric field gradient

Supplementary Figure 4 shows the evolution of the APT tip morphology during the simulation. The amplitude of the surface electric field from low to high is represented by the color codes from blue to red, respectively. The presence of a nanosized void is clear as it is exposed during field evaporation in Supplementary Figures 4b and 4c, and in a cross-section view of the tip when half of the void is exposed during evaporation (Supplementary Figure 4d).

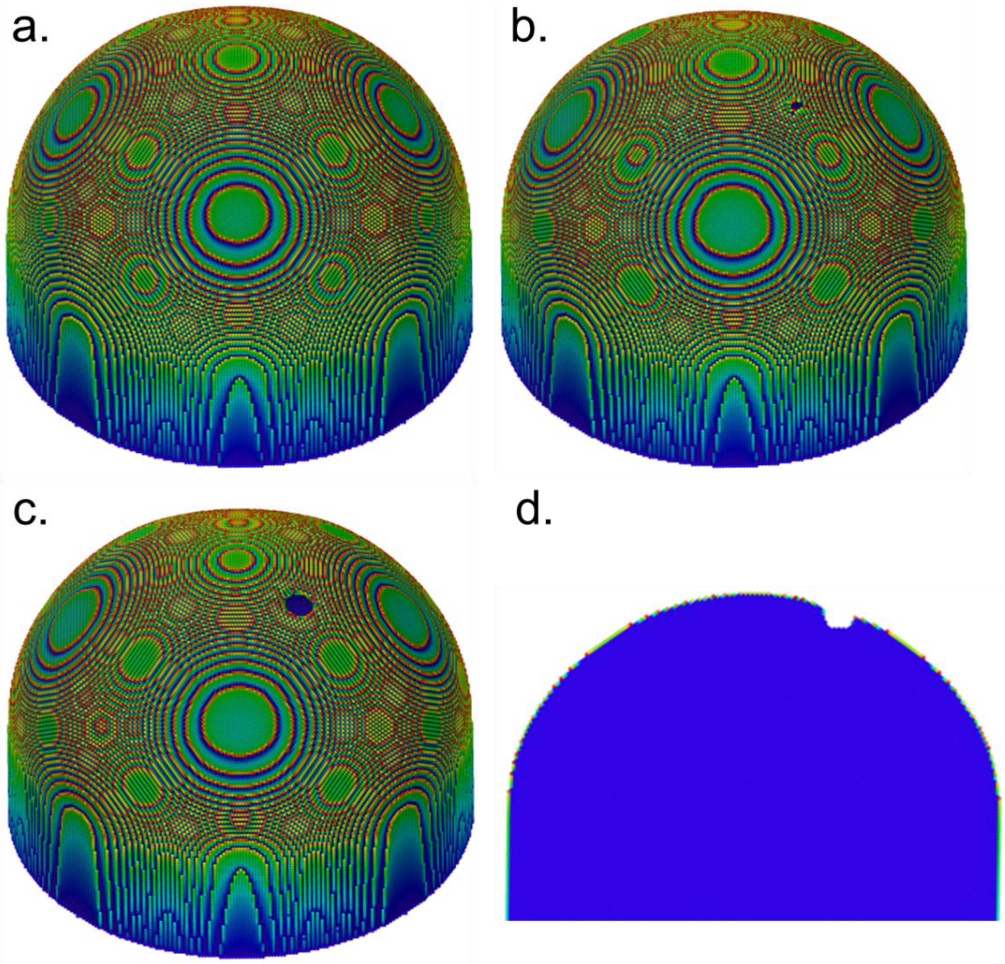

Supplementary Figure 4 Evolution of the APT tip morphology during the simulated APT experiment. (a-c) Tip morphology when the void is not evaporated, evaporated initially and half of the void is evaporated. The colour code represents the surface electric field amplitude; blue is low electric field and red is high electric field. (d) Cross-sectional view of the APT tip when half of the void is exposed during evaporation. This simulated tip is from a low-field shell case as discussed later.

Supplementary Note 5: Relation between shell evaporation fields and local density variations near nanovoids

Simulations with different evaporation fields of the segregation shell (E_{shell}) with respect to the matrix (E_{matrix}) have been performed with the results shown in Supplementary Figure 5. We varied E_{shell} from $0.2E_{matrix}$ to $2E_{matrix}$ and obtained the reduced density profile across the nanovoid. In Supplementary Figure 5, the y-axis is E_{shell}/ E_{matrix} and x-axis is the distance from the upper interface between the segregation shell and the matrix. Local reduced density values are represented by different colors. Based on the colormap,

the shape of density variations gradually evolve from the λ-shape to ω-shape as E_{shell} increases from 0.2 to $2E_{matrix}$. More quantitatively, when $E_{shell} < 0.8E_{matrix}$, the density variations show a characteristic λ-shape, when $E_{shell} > 1.1E_{matrix}$ the density variations show a characteristic ω-shape. In between these values, the density profile is a mixture of the λ and ω shapes. In the paper, $E_{shell} = 0.7E_{matrix}$ for the low-field case and $1.3E_{matrix}$ for the high-field case were chosen because and they are reasonable values which generated a good match to the experimental results; however, the λ and ω characteristic density variation shapes near nanovoids are not dependent on the magnitude of E_{shell} for $E_{shell} < 0.8E_{matrix}$ and $E_{shell} > 1.1E_{matrix}$.

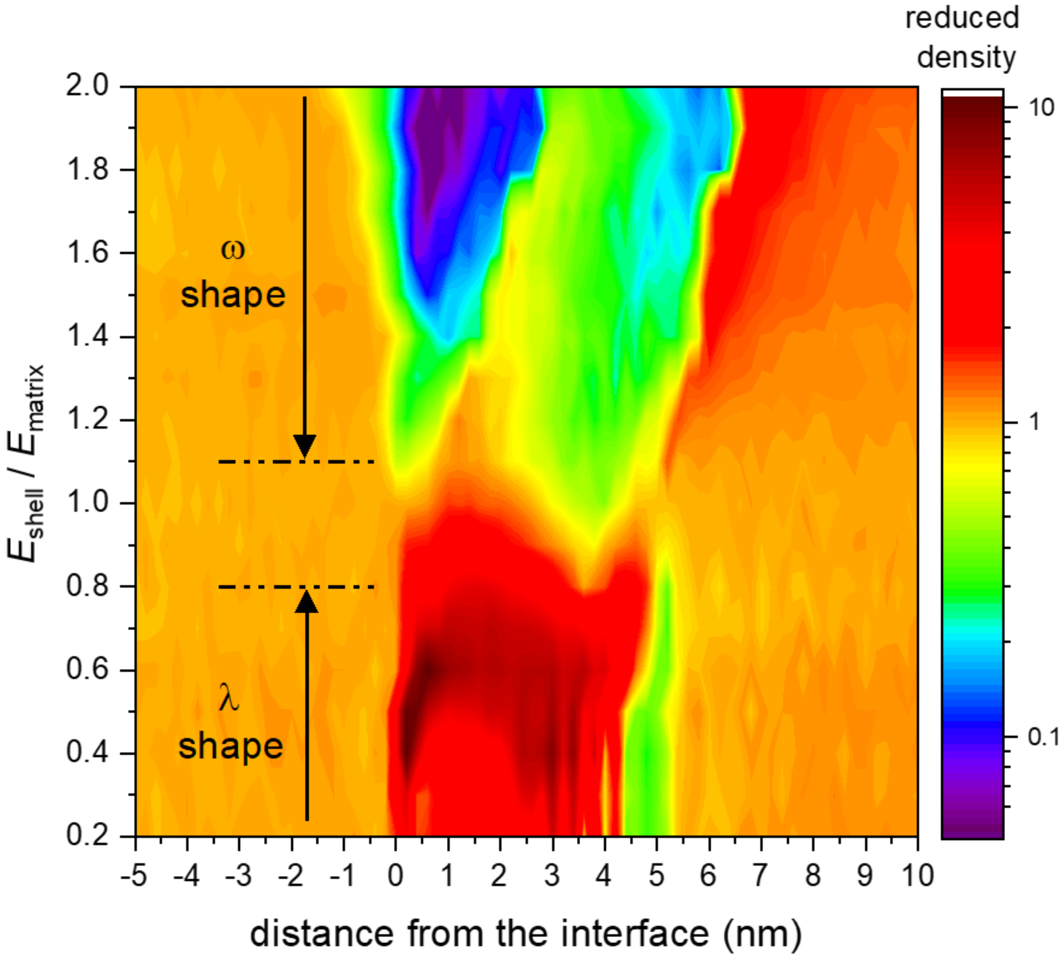

Supplementary Figure 5 Colormap showing local reduced density values near a nanovoid with different values for E_{shell}.

Supplementary Note 6: Si enrichment near cavities in doped NiCoCrFe

Despite the low doping concentration (less than 1 at. %), Si has a strong tendency to migrate to voids in the Si doped NiCoCrFe, which is shown in the 1D elemental concentration profile in Supplementary

Figure 6. The layer of enriched Si is most likely responsible for the increase in evaporation field of atoms near the void.

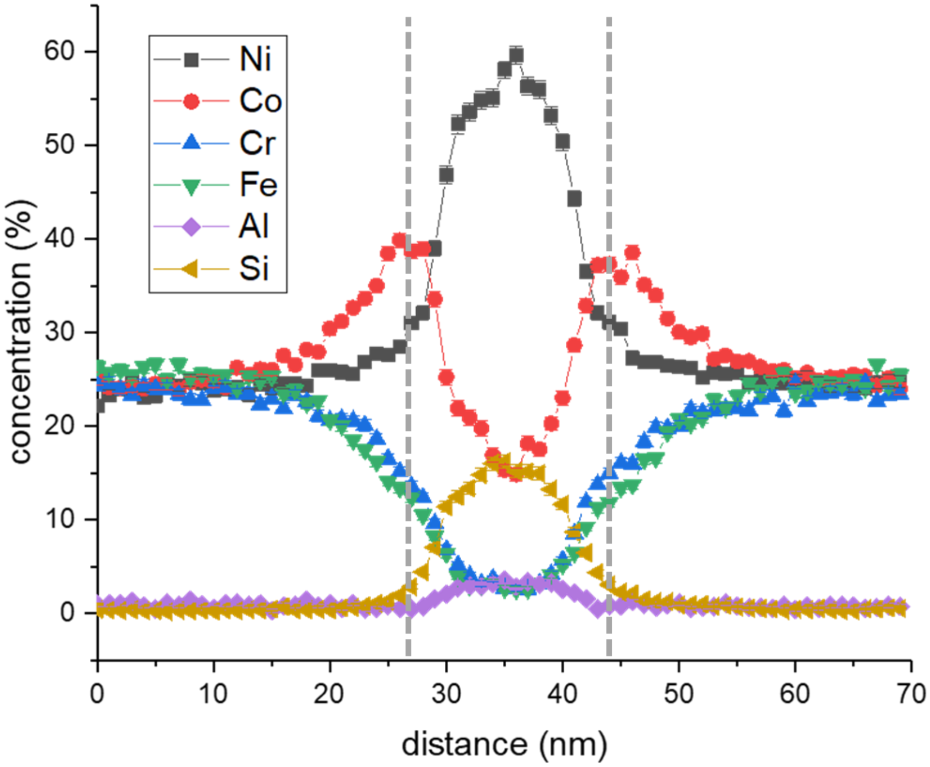

Supplementary Figure 6 1D concentration profile near a void in NiCoCrFe doped by Al and Si. The void position is marked by vertical dashed lines.

Supplementary Note 7: Overlapping mechanism in ring-shaped emitter

As mentioned in the main text, a ring-shaped emitter is generated when a void is open as the evaporation surface passes the void. The overlap from many small apexes on the ring can lead to an increase in hitting events on the 2D APT detector and an increase in local atomic density. Supplementary Figure 7 is a detector event histogram snapshot during a void evaporation, which shows the hitting event intensity is the highest in the center of the void. The inset in Supplementary Figure 7 is the calculated intensity profile from a ring-shaped illumination device, i.e. a ring light. Here we assume that ten illuminators are evenly distributed on the ring marked by the white dashed line, and each illuminator has a Gaussian light intensity distribution with the standard deviation equal to 1.2 times the ring radius. The calculated intensity profile shows a similar feature to the experimental detector event histogram, with highest intensity in the center of the ring. Note the comparison between the experimental detector event histogram and the inset is qualitative.

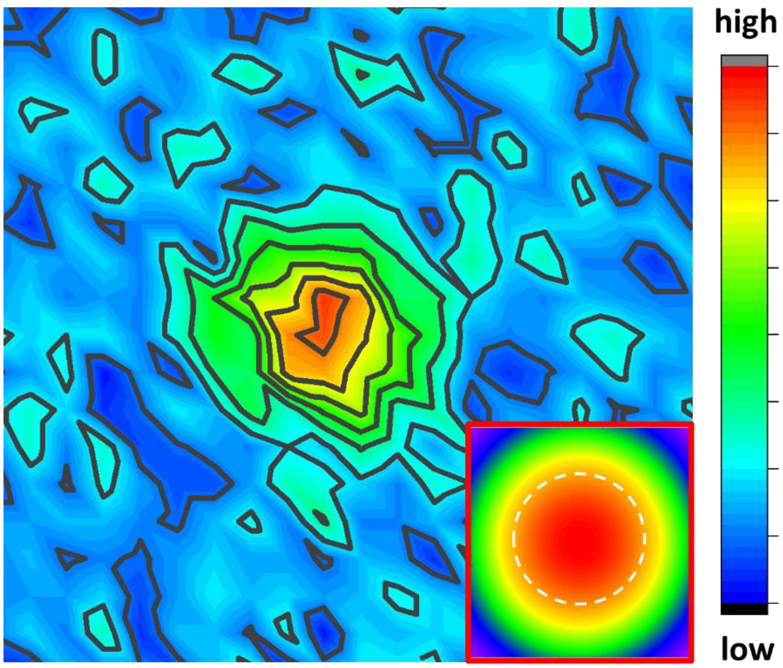

Supplementary Figure 7 An APT detector event histogram map captured during the evaporation of a void. The inset is the calculated intensity profile based on a ring-shaped illumination model.

Supplementary Note 8: Detailed analyses of ion trajectories near voids based on the simulated APT experiment

To illustrate the crossing of ion trajectories near the void, we plot a snapshot of the specimen tip surface (Supplementary Figures. 8a and b) and the corresponding hit positions of the atoms on the position-sensitive detector (Supplementary Figures. 8c and d) during the void evaporation process. In Supplementary Figure 8a, we color the atoms in rainbow color scale that represents the atom rotation angles with respect to the void center (ϕ), with red meaning ϕ equal to 0 and purple meaning ϕ infinitesimally close to 2π. The same atom projected onto the detector is in the same color as shown in Supplementary Figure 8c. It is clear that some purple atoms from the bottom left side of the void in Supplementary Figure 8a are projected onto the top right side of the void on the detector in Supplementary Figure 8c, and vice versa. This example explicitly shows the ion trajectory crossing introduced by the sharp rim of the void ring structure. In Supplementary Figure 8b, we color the atoms in rainbow color scale that represents the atom radial distances to the void center (r), with red meaning r is the farthest from and purple meaning r is the closest to the void center. The same atom projected onto the detector is in the same color as shown in Supplementary Figure 8d. It is clear that because of the abrupt increase in local magnification near the sharp rim of the ring structure, atoms closest to the void (purple) are projected farthest outward the void, and most atoms in the shell around

the void are concentrated on the void location on the detector, which should be empty if there is no ion trajectory aberration. The example shown in Figs. S8a-d is from the low-field simulation case.

To compare the ion trajectory aberrations in different evaporation fields and in different stages of void evaporation, we also provide the radial distance type plots in both the low-field (Supplementary Figures. 8e-h) and high-field simulations (Supplementary Figures. 8i-l) when half of the void is evaporated (Supplementary Figures. 8e, f, i, j) and when it is near the end of the field evaporation of the void (Supplementary Figures. 8g, h, k, l). Two trends can be observed using these plots. First, comparing Supplementary Figures. 8f and j, we find that the ion trajectory aberrations are larger in the low-field case than the high-field case. Second, comparing Supplementary Figures. 8f and h, or j and l, we can find that in both the low and high-field cases, the ion trajectory aberrations become smaller as the void evaporation comes to the end. This trend supports our conclusion in the main text that a more accurate segregation measurement can be obtained near the bottom region of the void in the APT reconstruction.

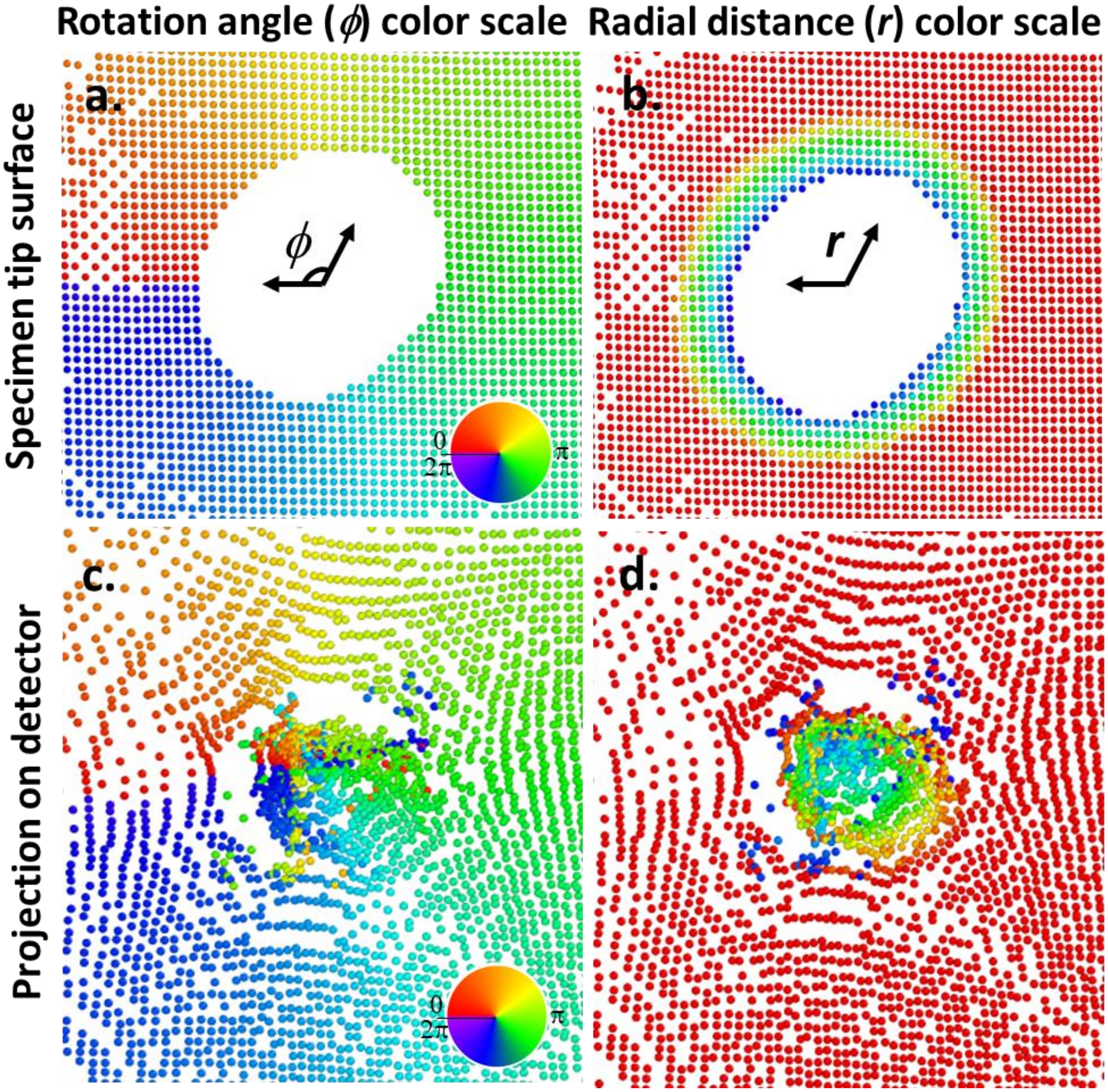

Rotation angle (ϕ) color scale
Radial distance (r) color scale
Specimen tip surface
Projection on detector
a.
b.
c.
d.
ϕ
r
0
2π
π

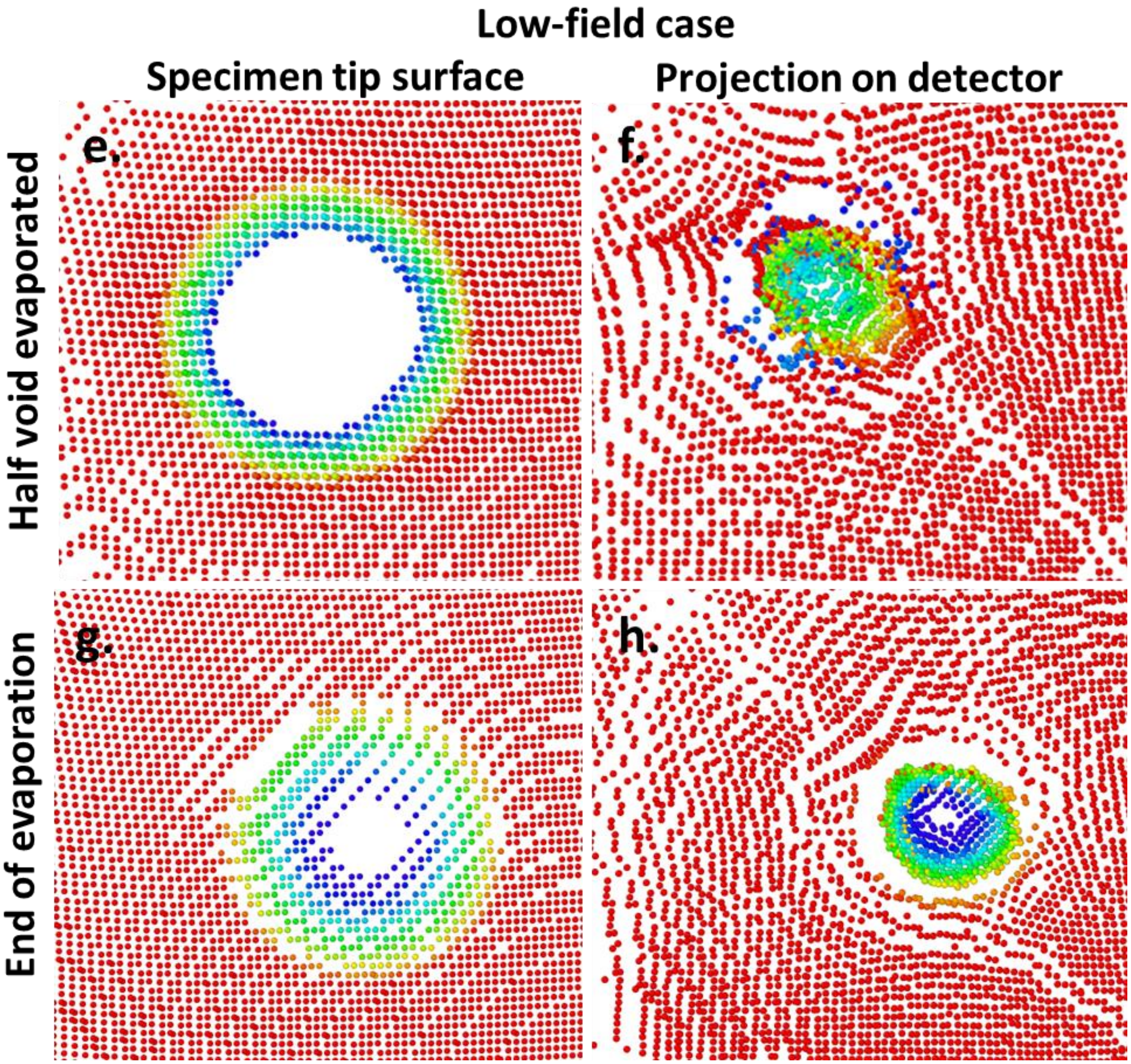
Low-field case
Specimen tip surface
Projection on detector
Half void evaporated
End of evaporation
e.
f.
g.
h.

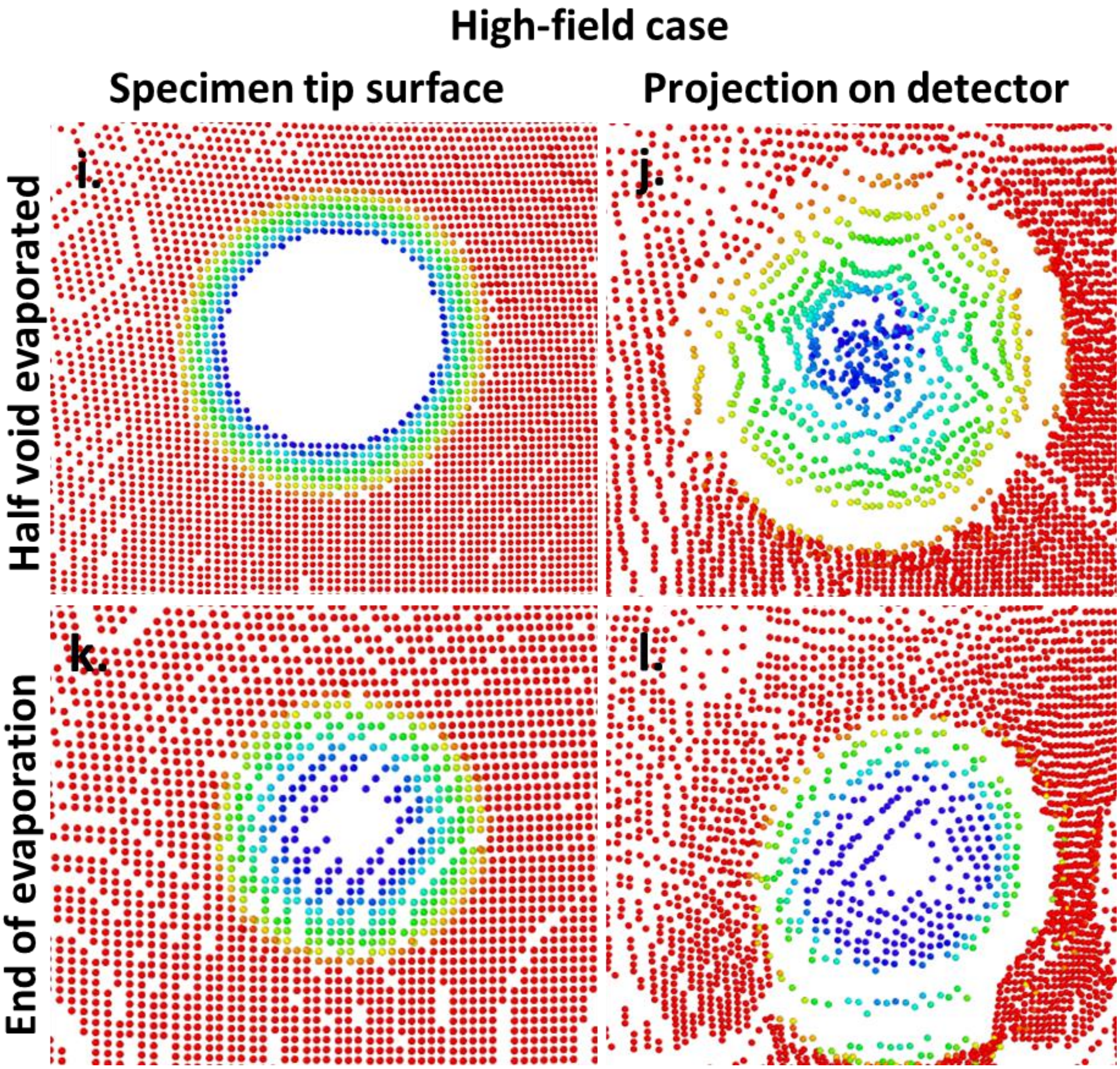

Supplementary Figure 8 Snapshot of the simulated APT tip surface and its corresponding atom hitting positions on the position-sensitive detector. (a) and (c), (b) and (d), (e) and (f), (g) and (h), (i) and (j), (k) and (l) are the tip surface in plain view and their corresponding detector hitting positions, respectively. In (a) and (c), atoms are colored in rainbow scale representing their original rotation angles with respect to the void center; in other figures, atoms are colored in rainbow scale representing their original distance to the void center.

Supplementary Note 9: Transmission electron microscopy (TEM) analysis of radiation-induced voids in Ni and Ni-based single phase concentrated solid solution alloys (SP-CSAs)

Bright field (BF)-TEM images were obtained from the samples containing nano-voids. As these BF-TEM images were taken using an under-focused condition, voids appear as high intensity regions. For the Ni, NiFe and NiCoCr samples irradiated by 200 keV He ions, the BF-TEM were acquired at about 50 nm to 350 nm below the surface (Supplementary Figures. 9a, b, c); for the doped NiCoCrFe sample irradiated by 3 MeV Ni ions, the BF-TEM images were acquired at about 1100 nm to 1450 nm below the surface (Supplementary Figure 9d). There regions are where the nano-voids mostly reside in the irradiated materials and where the needle-shaped APT specimens were prepared.

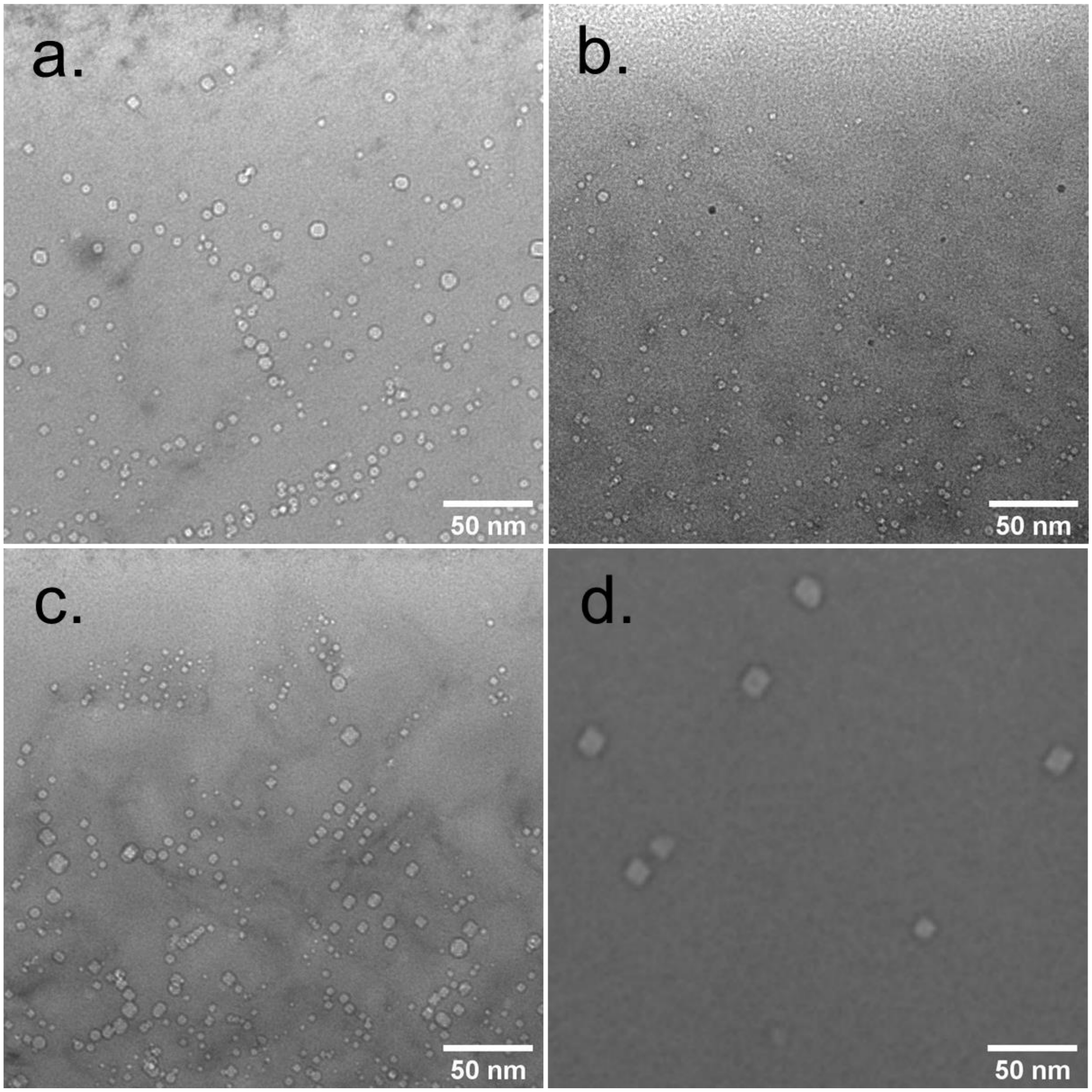

Supplementary Figure 9 Bright field (BF)-TEM images shown void size and distribution in different samples at under-focused condition. (a) Ni. (b) NiFe. (c) NiCoCr. (d) Doped NiCoCrFe. Voids exhibit as bright circles in these under-focused BF-TEM images.

Supplementary Note 10: 1D density profiles from all voids shown in Fig. 1

In Supplementary Fig. 10, we plot the 1D density profiles of each voids shown in Fig. 1 to demonstrate the observed characteristic density profile is not a specific case for certain voids. Supplementary Figure 10a shows the void positions in the APT reconstruction and 10b shows the local density variations near voids. It is clear that different void sizes lead to different magnitudes of local density increase, but all the density profiles near nanovoids in NiCoCr exhibit a characteristic λ-shape. Note void 2 in Supplementary Figure 10a is not shown in 10b because this void is intercepted by the APT specimen surface, so its local density variation is altered.

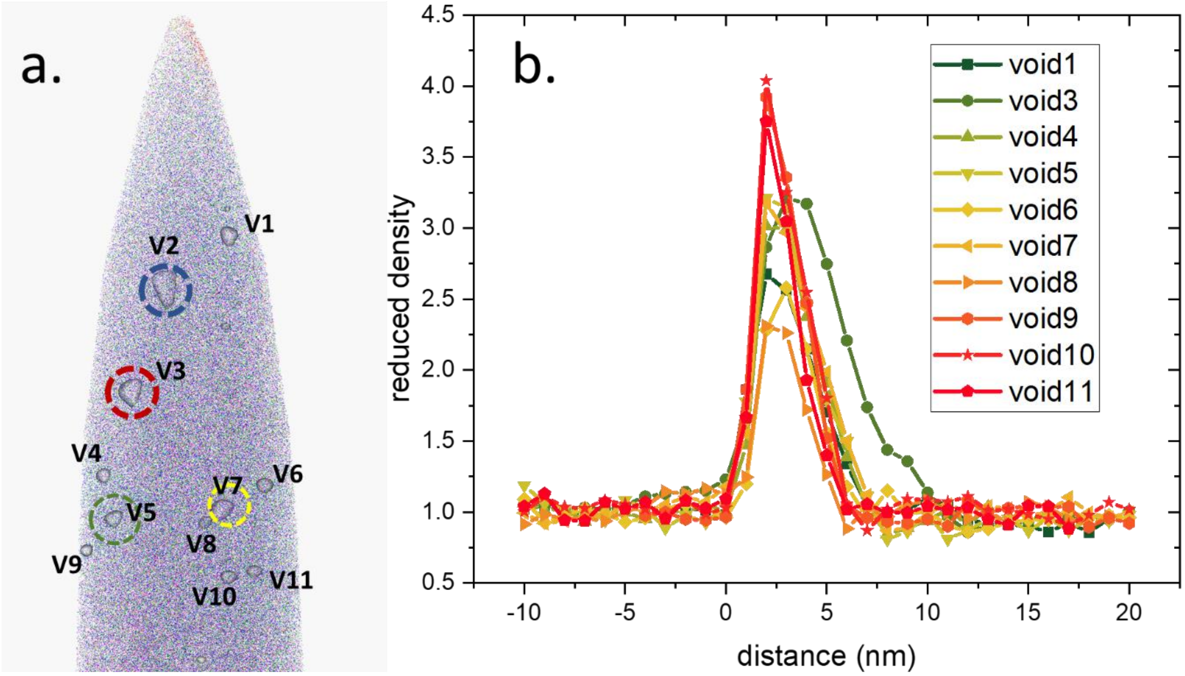

Supplementary Figure 10 Local density profiles from multiple voids in NiCoCr sample. (a) APT reconstruction showing void locations and labels. (b) 1D Density profiles passing through each void as shown in (a). The reduced density is the local atomic density divided by the average atomic density in the matrix. For the x-axis, lower distance value indicates the region is evaporated earlier during the APT experiment and the position where local density starts to increase is defined as zero. Note the APT reconstruction in (a) is the same as that in Fig. 1g but with a smaller image magnification.
