# Supplementary material for: Interpreting nanovoids in atom probe tomography data for accurate local compositional measurements
Source: Nat Commun. 2020 Feb 24;11:1022. doi: 10.1038/s41467-020-14832-w (PMC7039975; doi:10.1038/s41467-020-14832-w)
Supplement: Supplementary file 3 — Description of Additional Supplementary Information [file 41467_2020_14832_MOESM3_ESM.pdf]

**Description of Additional Supplementary Files**

File Name: Supplementary Movie 1
Description: Rotation series of the same needle-shaped specimen from the APT reconstruction and STEM images. In the APT reconstruction on the left, voids are represented by white high density iso-surfaces. In the STEM-HAADF images on the right, voids are exhibited as low intensity regions.

File Name: Supplementary Movie 2
Description: Marked rotation series of the same needle-shaped specimen from the APT reconstruction and STEM images. The same void is marked by circles in the same color in both the APT reconstruction and the STEM-HAADF image.

File Name: Supplementary Movie 3
Description: Simulated evolutions of tip surface morphology during the void field evaporation process. The surface morphology evolutions for the tip containing voids with a high evaporation field shell (left) and a low evaporation field shell (right). Shell curvature near the void is higher than the rest of the tip surface in the high-field case and is lower in the low-field case.
